# Pulmonary Hypertension-Associated Right Ventricular Cardiomyocyte Remodelling Reduces Treprostinil Function

**DOI:** 10.3390/cells12232764

**Published:** 2023-12-04

**Authors:** Aleksandra Judina, Marili Niglas, Vladislav Leonov, Nicholas S. Kirkby, Ivan Diakonov, Peter T. Wright, Lan Zhao, Jane A. Mitchell, Julia Gorelik

**Affiliations:** 1 Cardiac Section, National Heart and Lung Institute (NHLI), Faculty of Medicine, Imperial College London, Hammersmith Campus, Du Cane Road, London W12 0NN, UK; a.judina18@imperial.ac.uk (A.J.); m.niglas18@imperial.ac.uk (M.N.); vleonov@medicine.wisc.edu (V.L.); n.kirkby@imperial.ac.uk (N.S.K.); i.diakonov@imperial.ac.uk (I.D.); l.zhao@imperial.ac.uk (L.Z.); j.a.mitchell@imperial.ac.uk (J.A.M.); 2 Department of Surgery, Dentistry, Pediatrics and Gynecology, Cardiovascular Science, The University of Verona, 37134 Verona, Italy; 3 Definitely School of Life and Health Sciences, Whitelands College, University of Roehampton, Holybourne Avenue, London SW15 4JD, UK; peter.wright@roehampton.ac.uk

**Keywords:** pulmonary hypertension, right ventricle, cardiomyocytes, treprostinil, sarcomere shortening, cell length deflection

## Abstract

(1) Pulmonary hypertension (PH)-associated right ventricular (RV) failure is linked to a reduction in pulmonary vasodilators. Treprostinil has shown effectiveness in PAH patients with cardiac decompensation, hinting at potential cardiac benefits. We investigated treprostinil’s synergy with isoprenaline in RV and LV cardiomyocytes. We hypothesised that disease-related RV structural changes in cardiomyocytes would reduce contractile responses and cAMP/PKA signalling activity. (2) We induced PH in male Sprague Dawley rats using monocrotaline and isolated their ventricular cardiomyocytes. The effect of in vitro treprostinil and isoprenaline stimulation on contraction was assessed. FRET microscopy was used to study PKA activity associated with treprostinil stimulation in AKAR3-NES FRET-based biosensor-expressing cells. (3) RV cells exhibited maladaptive remodelling with hypertrophy, impaired contractility, and calcium transients compared to control and LV cardiomyocytes. Combining treprostinil and isoprenaline failed to enhance inotropy in PH RV cardiomyocytes. PH RV cardiomyocytes displayed an aberrant contractile behaviour, which the combination treatment could not rectify. Finally, we observed decreased PKA activity in treprostinil-treated PH RV cardiomyocytes. (4) PH-associated RV cardiomyocyte remodelling reduced treprostinil sensitivity, inotropic support, and impaired relaxation. Overall, this study highlights the complexity of RV dysfunction in advanced PH and suggests the need for alternative therapeutic strategies.

## 1. Introduction

In pulmonary hypertension (PH), dysfunction of the right heart is a major factor affecting patient prognoses [1]. PH results from hormonal imbalances and endothelial dysfunction, leading to chronic obstructive changes in pulmonary arteries and rapid maladaptive remodelling of the right ventricle (RV), often culminating in right heart failure [2,3,4,5]. Recent data from PH patients indicate high mortality rates, especially in intermediate- to high-risk individuals [6].

The healthy right ventricular (RV) myocardium exhibits high compliance and only generates about 20% of the cardiac energy output. In response to pathologically elevated pulmonary vascular resistance (PVR) [5,7], the RV undergoes rapid dilation to compensate [8]. Both isovolumetric contraction and relaxation of the dilated RV are increased, elevating the basal oxygen consumption; however, it has limited adaptation potential compared to the LV [9]. 

Cardiomyocyte contraction is regulated by neurohumoral signalling via β-adrenergic receptor (β-AR) activation that modulates intracellular cAMP pools and kinase A (PKA) activity at micro-domain levels [10]. The role of chronic sympathetic stimulation is well-studied in LV failure [11], while the mechanism of RV dysfunction in pulmonary arterial hypertension (PAH) remains less clear [12,13,14]. A recent comparison between PH patients and those with LV failure with reduced ejection fraction revealed that sympathetic nervous system hyperactivity significantly contributes to maladaptive RV remodelling [15].

Treprostinil is a stable PGI_2_ mimetic drug which is used to manage PAH and has shown therapeutic potential, with treatment doubling the 3-year survival rate of PAH patients [3,16,17]. Furthermore, pharmacological assessment of the drug targeting revealed specific targeting of the right heart, compared to the lungs and LV [18,19].

To study the effect of treprostinil on failing RV cardiomyocytes, we employed a well-established rat model of established pulmonary hypertension (PH) induced by monocrotaline (MCT), which mimics the RV hypertrophy and failure seen in PH [20,21]. MCT-treated animals exhibit compensatory RV remodelling associated with Ca^2+^ signalling alterations [22]. Three weeks post MCT, animals reach the end-stage of RV maladaptive remodelling, and exhibit the decompensation and rHF [20,21] resembling the features of PAH functional classes III and IV [23]. 

Our study aimed to assess the physiological and morphological changes in PH RV cardiomyocytes and whether treprostinil affects their contractility by modulating catecholamine-induced cAMP/PKA signalling. 

## 2. Materials and Methods

### 2.1. Reagents

All reagents were purchased from Sigma-Aldrich, Gillingham, UK, unless otherwise specified.

### 2.2. Animal Ethics

This work was carried out under the regulations of Imperial College London and in compliance with the UK Animal in Scientific Procedures Act (ASPA) 1986 and the 2010/63/EU Directive, which conforms to the Guide for the Care and Use of Laboratory Animals published by the US National Institute of Health in 1996.

### 2.3. Generation of the MCT-Induced Rat PH Model

Adult male Sprague Dawley rats were purchased from Charles River (UK) and housed individually in a temperature-controlled room on a 12 h light/dark cycle, with food and water provided ad libitum. Animals were randomly assigned to PH (*n* = 5) or age-matched vehicle-treated control (Sham) (*n* = 5) groups, and the model was induced as previously described [20,21]. Briefly, rats ranging in body weight (BW) from 18 to 220 g received a single subcutaneous (S.C.) injection of 60 mg/kg of MCT (PH) or vehicle (0.9% normal saline; 1.5 mL/kg) (Sham). BW was monitored daily, and rats were sacrificed three weeks post-injection. Heart weight (HW), lung weight (LW), and tibia length (TL) were measured post-mortem. 

### 2.4. Ventricular Cardiomyocyte Isolation and Culture

RV and LV cardiomyocytes from PH and Sham animals were isolated 21–23 days post-injection using a standard protocol involving retrograde Langendorff perfusion and enzymatic digestion [24]. Isolated RV cardiomyocytes were centrifuged at 1000 RPM for 1 min and resuspended in Imaging Solution [25] or MEM media (1% AA, 1% L-glutamine, 9 mM NaHCO_3_) [26] supplemented with 10% foetal bovine serum (FBS). RV cardiomyocytes were plated at 100 cells/μL on laminin-coated 35 mm glass-bottom dishes (MatTek, Ashland, OR, USA) (for Contraction studies) or on a 25 mm glass coverslips (for FRET Microscopy) in MEM media. Plated RV cardiomyocytes were incubated for 1 h at room temperature (contraction study) or 37 °C, 5% CO_2_ (FRET microscopy). 

### 2.5. Assessment of Ventricular Cardiomyocyte Morphological Changes

A CytoCypher–high-throughput system (HTS) (IonOptix, Westwood, MA, USA) was used to record thumbnail images of resting, freshly isolated cardiomyocytes from PH and Sham animals. Two-dimensional morphological (cell minor (width) and major axis (length)) and spectral information (relative power of sarcomere regularity based on the frequency component of a Fast Fourier Transform (FFT) 1.8 µm) were derived from the cells as previously described [25]. A bespoke macro (Fiji ImageJ, v 2.0.0-rc-69/1.52p) [25] generated cell segment masks. These were used to drive the analysis of the original images using CytoSpectre [27] run via MatLab (R2021b, v9.11.0.1769968, maci64). 

### 2.6. Measuring Cell Contraction Using CytoCypher–HTS

Cardiomyocytes were exposed to 1 μM of Fura-2AM for 45 min, followed a 15 min wash with Imaging Solution [25]. Cells were paced at 1 Hz and left for 5 min before acquiring the data to allow for the normalisation of contraction. RV cardiomyocyte Sarcomere Shortening, Cell Length Deflection, and calcium transients (CaTs) were recorded for ~10 cells per plate using the CytoSolver Transient Analysis Tool (IonOptix LLC, Westwood, MA, USA). The cells were then treated with 1 μM of treprostinil (a clinically relevant concentration [28]), 1μM of isoprenaline [26] or a combination of both drugs. The CytoCypher–HTS system automatically detected the proportion of arrhythmic cells. Cardiomyocyte arrhythmia was assessed based on the CaT and cellular contractility focusing on any deviations from the 1 Hz pacing frequency. 

### 2.7. Measuring Nuclear cAMP/PKA Levels Using Multi-Cell FRET Microscopy

RV cardiomyocytes from PH and Sham animals were cultured in MEM media at 37 °C 5% CO_2_ and were infected with an adenoviral vector leading to the expression of the A-kinase activated reporter–nuclear-localised sensor (AKAR-NLS) [29] (MOI 1000 pfu/cell) FRET-based construct. The FRET probe consisted of CFP and YFP fluorophores with their exchange energy activated by PKA phosphorylation of the 14–3-3τ sensor target sequence [30].

At 48 h post-infection, the cardiomyocytes were washed with FRET buffer solution (144 mM NaCl; 16 mM HEPES; 5 mM KCl; 1 mM MgCl_2_; pH 7.2–7.3) and treated with incrementally increasing concentrations of treprostinil (0.01 μM, 0.1 μM, 1 μM, and 10 μM) followed by saturator (10 μM forskolin, 100 μM IBMX) [31]. After the FRET ratio response plateau was established, each subsequent pharmacological treatment was added. Throughout the measurement, images of the cells were acquired to quantify relative CFP and YFP emissions using ratio-metric multi-cell FRET microscopy every 12 s and were analysed as previously described [32]. 

### 2.8. Statistics

The data were analysed using Prism 9 Software v9.3.1 and are reported as mean ± standard error of the mean (SEM) for *n* (number of animals or cells) (two-way ANOVA only), and a *p* < 0.05 was considered statistically significant. The statistical tests are reported in the figure legends. Normality was assessed using the Shapiro–Wilk test for PH and Sham rat data, and the Kolmogorov–Smirnov test was applied for isolated cardiomyocyte data. A non-parametric Mann–Whitney test was used to compare PH and Sham rats. Nested data analysis was applied as previously recommended to compare single cell contraction and CaT recordings of PH and Sham cardiomyocytes [33]. Nested one-way ANOVA with Tukey’s Multiple Comparisons was applied to compare pharmacological treatment-induced effects. A two-way ANOVA with Šídák’s multiple comparisons was used to evaluate the changes in FRET signalling induced by treprostinil, which are linked to the activity of nuclear PKA.

## 3. Results

### 3.1. Rats with Established PH Exhibit Increased Heart and Lung Weights 3 Weeks Post-MCT Treatment

**Figure 1:**
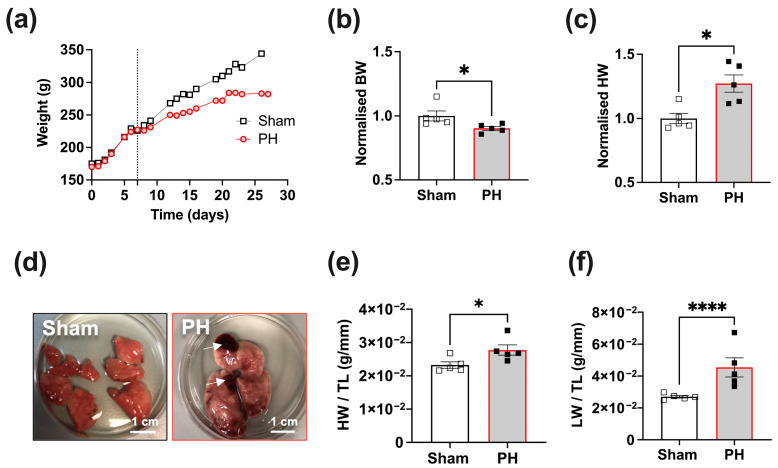
Characterisation of pulmonary hypertension (PH) and Sham rats. (**a**) Representative traces of the body weight changes in PH and Sham rats. The dotted line indicates the time point of MCT or vehicle administration. (**b**) Normalised body weight (BW) of PH and Sham rats. (**c**) Normalised heart weight of PH and Sham rats. (**d**) Representative images of explanted lungs from PH (red box) and Sham (black box) rats post-treatment. The lungs from PH animals exhibited lung damage (indicated with white arrows). (**e**) Heart weight (HW)/tibia length (TL) ratios of PH and Sham rats. (**f**) Lung weight (LW)/TL ratios of PH and Sham rats. PH animals (black square); Sham animals (white square). Data presented as mean ± SEM, *n* = 5; * *p* < 0.05, **** *p* < 0.0001, by Mann–Whitney test.

In our experiments, we utilised an MCT-induced PH model to assess RV failure-associated pathophysiological changes in RV and LV cardiomyocytes. Three weeks post-treatment, PH animals displayed reduced body weight (BW) compared to Sham animals (Figure 1a).

We characterised PH and Sham rats’ cardiothoracic organs post mortem. A comparison of the mean normalised BW revealed a significant decrease in the PH group, with an average 10% reduction (*p* < 0.05, Figure 1b) compared to the Sham group. PH animals also exhibited a significant increase in the normalised heart weight (HW), with a 27% increase (*p* < 0.05, Figure 1c). All PH animals exhibited variable degrees of macroscopic signs of pulmonary tissue damage, identified visually as dark infarct-like areas (Figure 1d). HW and lung (LW) weights were normalised to tibia length (TL) to confirm pulmonary and cardiac remodelling. In comparison to the Sham group, the PH animals demonstrated a significant increase in the HW/TL ratio, indicative of pronounced cardiac remodelling associated with the pathology.

### 3.2. RV Cardiomyocytes from Rats with Established PH Exhibit Hypertrophy and Reduction in Sarcomere Regularity

**Figure 2:**
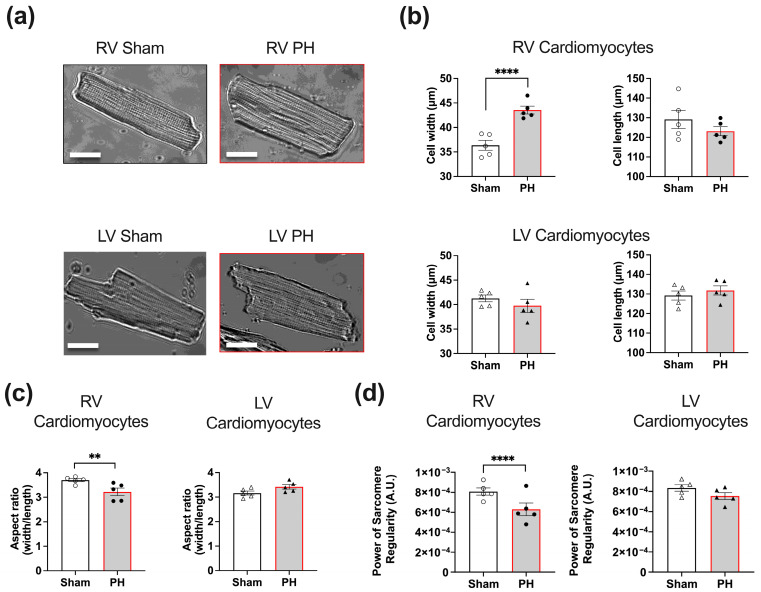
Characterisation of RV and LV cardiomyocytes from pulmonary hypertension (PH) and Sham animals. (**a**) Representative images of the PH and Sham RV and LV cardiomyocyte; scale bar = 20 μm. Mean cardiomyocyte cell width and length (**b**), aspect ratio (**c**), and power of sarcomere regularity (**d**). PH RV cardiomyocytes (black dots), PH LV cardiomyocytes (black triangles), Sham RV cardiomyocytes (white dots), Sham LV cardiomyocytes (white tringles). Data presented as mean ± SEM, *n* = 5; ** *p* < 0.01, **** *p* < 0.0001 by nested *t*-test.

We used the images of the un-paced PH and Sham RV and LV cardiomyocytes (Figure 2a) to assess the 2D cell morphology and sarcomere regularity. We found that PH pathology significantly increased the RV cardiomyocyte cell width (43.138 ± 1.054 μm vs. 36.266 ± 0.9465 μm, *p* < 0.0001, Figure 2b). The PH RV and LV cardiomyocyte cell length and PH LV cardiomyocyte cell width did not significantly differ from Sham cells (Figure 2b). The aspect ratio of the PH RV cardiomyocytes was significantly elevated (3.277 ± 0.067 vs. 3.695 ± 0.09, *p* < 0.01, Figure 2c) compared to Sham, and no effect was observed in the LV cardiomyocyte populations. 

We also found a significant decrease in the power of PH RV cardiomyocyte sarcomere regularity (6.295 × 10^−4^ ± 1.538 × 10^−5^ vs. 8.016 × 10^−4^ ± 3.031 × 10^−5^, *p* < 0.0001, Figure 2d) compared to Sham RV cells, while the LV cardiomyocytes displayed no significant alteration.

### 3.3. RV Cardiomyocytes from Rats with Established PH Exhibit Impaired Contraction and Ca^2+^ Transients (CaTs)

**Figure 3:**
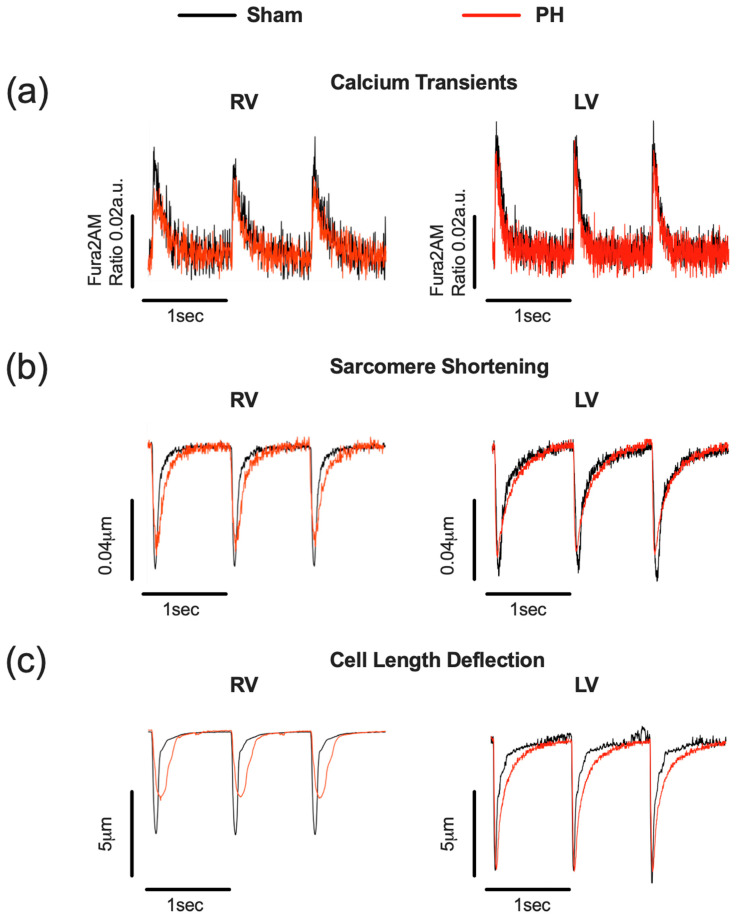
Remodelling of ventricular cardiomyocyte contraction and calcium transients (CaTs) in pulmonary hypertension (PH). Representative calcium transients (**a**), Sarcomere Shortening (**b**), and Cell Length Deflections (**c**) in PH (red) and Sham (black) right (RV) and left (LV) ventricular cardiomyocytes.

We compared the PH and Sham ventricular cardiomyocyte CaT, Sarcomere Shortening, and Cell Length Deflection (Figure 3 and Table 1) parameters at baseline. PH pathology was associated with significant CaT alterations in RV but not LV cardiomyocyte populations (Figure 3a and Table 1). RV cardiomyocytes displayed a significant reduction in peak mean Ca^2+^ amplitude, an increase in time taken to reach 90% of the peak (TTP90) and a prolongation of time taken to reach 90% of the baseline (TTB90) compared to Sham cells (Table 1). No significant changes in CaTs were identified in the PH LV cardiomyocyte population compared to Sham cardiomyocytes (Table 1). 

Next, the baseline parameters of RV and LV cardiomyocyte contractions from Sham and PH animals were assessed (Figure 3b, Table 1). PH RV cardiomyocytes displayed a significantly lower percentage of Sarcomere Shortening, prolonged TTP90, and prolonged TTB90 (Table 1) compared to Sham. PH LV cardiomyocytes also exhibited a significant increase in TTP90 and TTB90 (Table 1).

The Cell Length Deflection of PH RV and LV cardiomyocytes also exhibited alterations (Figure 3c and Table 1). The PH RV cardiomyocytes exhibited a decreased percentage Cell Length Deflection, prolonged TTP90, and prolonged TTB90 (Table 1). In LV cardiomyocytes, there was a trend for an increased percentage of Cell Length Deflection and a significant reduction in TTP90 compared to Sham LV cardiomyocytes (Table 1).

### 3.4. RV Cardiomyocytes from PH Animals Exhibit a Reduced CaT

**Figure 4:**
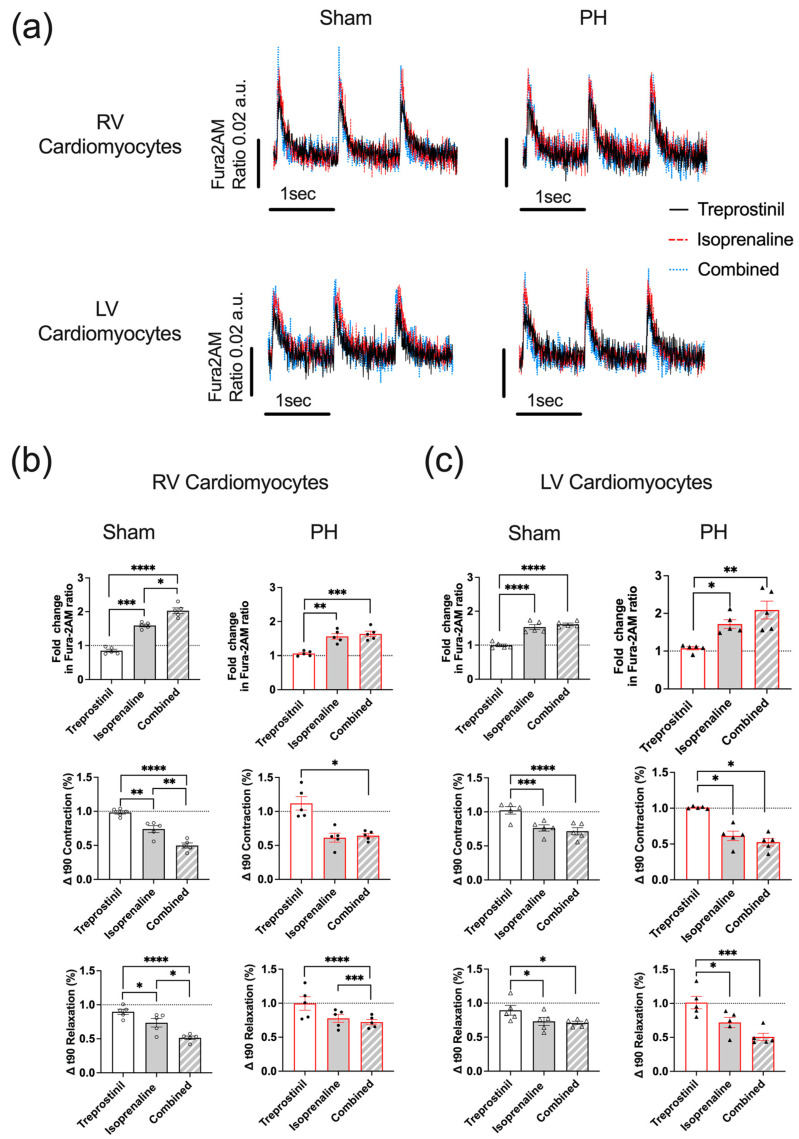
Characterisation of the effects of treprostinil, isoprenaline, and the combination of treprostinil and isoprenaline on Ca^2+^ transients (CaTs) in PH ventricular cardiomyocyte populations. (**a**) Representative traces of CaTs in RV and LV cardiomyocytes treated with treprostinil (black solid line), isoprenaline (red dashed line), and the combination of treprostinil and isoprenaline (blue dotted line). Treatment-associated normalised mean CaT parameters of PH (red) and Sham (black) RV the (**b**) and LV (**c**) cardiomyocytes. PH RV cardiomyocytes (black dots), PH LV cardiomyocytes (black triangles), Sham RV cardiomyocytes (white dots), Sham LV cardiomyocytes (white tringles). Data expressed as mean ± SEM; *n* = 5 rats; * *p* < 0.05, ** *p* < 0.01, *** *p* < 0.001, **** *p* < 0.0001 based on one-way ANOVA with Tukey’s multiple comparisons.

We next investigated the effect of acute treprostinil, isoprenaline, and the combination of both drugs on RV and LV cardiomyocyte CaTs (Figure 4a). We first assessed the impact of treprostinil on CaTs and observed that treprostinil treatment did not influence CaTs in PH RV cells. In PH RV cells, isoprenaline was able to induce a 1.5-fold increase in the Fura-2AM ratio, and about a 40% decrease in the TTP90 normalised to baseline (Δt90 Contraction) and a 20% decrease in TTB90 normalised to baseline (Δt90 Relaxation). In Sham cardiomyocytes, isoprenaline induced a 1.6-fold increase in the Fura-2AM ratio and about a 30% decrease in Δt90 Contraction and Δt90 Relaxation compared to baseline. The combination of treprostinil and isoprenaline in PH cardiomyocytes did not alter the CaT (Figure 4b) or proportion of arrhythmic cells (Figure S1a,b) compared to an effect observed for isoprenaline stimulation. In Sham cells, the combination of treprostinil and isoprenaline caused a significant increase in the Fura-2 ratio fold change (2.030 ± 0.084 vs. 1.592 ± 0.041, *p* < 0.05), and a significant decrease in Δt90 Contraction (0.498 ± 0.037 vs. 0.739 ± 0.050, *p* < 0.01) and Δt90 Relaxation (0.515 ± 0.026 vs. 0.736 ± 0.063, *p* < 0.05) compared to isoprenaline (Figure 4b).

In LV cardiomyocytes, isoprenaline induced a significant 1.7-fold increase in the PH and a 1.5-fold increase in the Sham LV cardiomyocyte Fura-2AM ratios. Isoprenaline also caused a 30% reduction in Δt90 Contraction and Δt90 Relaxation, while in Sham LV cardiomyocytes, there was only a 25% reduction in both parameters (Figure 4c). The combination of treprostinil and isoprenaline induced a 2.1-fold increase in the Fura2-AM ratio in PH LV cardiomyocytes, but it was not statistically significant compared to the effect of isoprenaline. There was no further change in CaTs caused by treprostinil and isoprenaline compared to the effect of isoprenaline alone in Sham LV cells (Figure 4c). In LV cardiomyocytes, the combination of treprostinil and isoprenaline did not cause a significant change in the proportion of arrhythmic cells compared to the effect observed for isoprenaline stimulation (Figure S1a,c).

In addition, there was a 30% reduction in Δt90 Contraction and a 50% reduction in Δt90 relaxation in PH LV cardiomyocytes following the treatment with the combination of the two drugs. There was a 25% reduction in both parameters in Sham cells, although the effects caused by isoprenaline and the combination of isoprenaline and treprostinil were not statistically significant.

### 3.5. Potentiation of the Isoprenaline Positive Inotropic Effect by Treprostinil Is Lost in PH RV Cardiomyocytes

**Figure 5:**
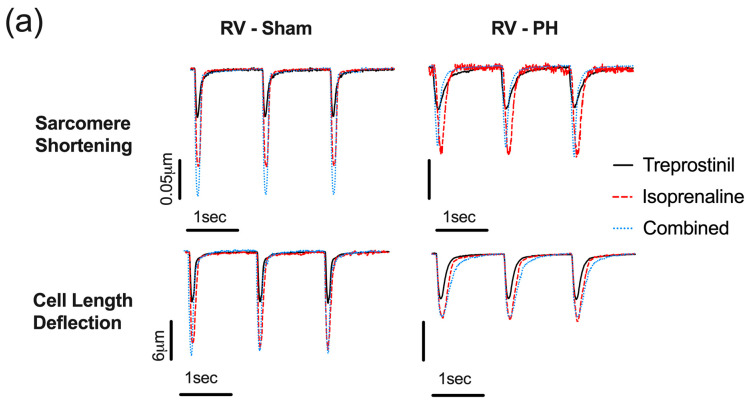

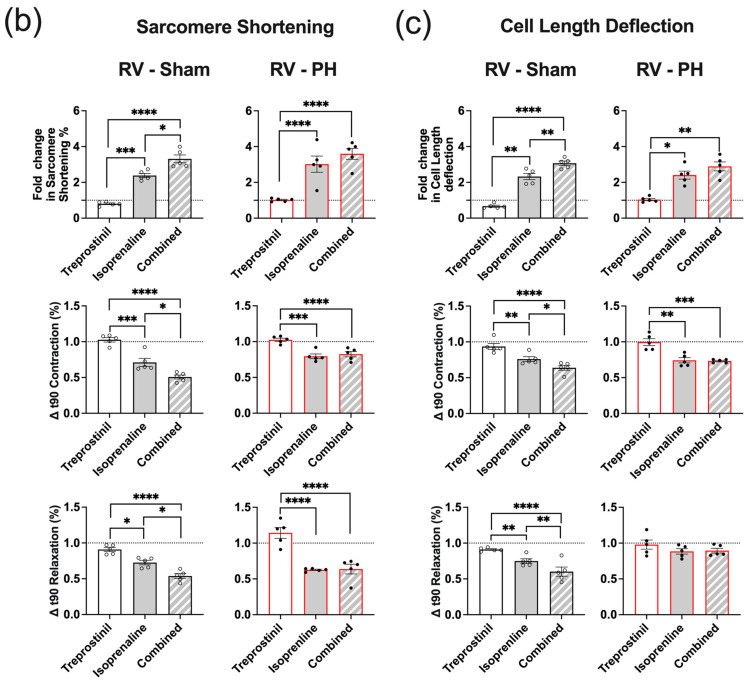
Characterisation of effects of treprostinil, isoprenaline, and the combination of treprostinil and isoprenaline on PH (red) and Sham (black) RV cardiomyocytes. Representative traces (**a**) and normalised mean percentage Sarcomere Shortening (**b**) and Cell Length Deflection (**c**). PH RV cardiomyocytes (black dots), Sham RV cardiomyocytes (white dots). Data expressed as mean ± SEM, *n* = 5 rats; * *p* < 0.05, ** *p* < 0.01, *** *p* < 0.001, **** *p* < 0.0001 by nested one-way ANOVA with Tukey’s multiple comparisons.

The next set of experiments examined the effect of treprostinil, isoprenaline, and the combination on Sarcomere Shortening and Cell Length Deflection (Figure 5). The representative traces shows that there were drug-associated contractility changes compared to Sham cardiomyocytes (Figure 5a).

The treprostinil treatment had no observable effect on PH RV cardiomyocytes Sarcomere Shortening, while in Sham cells, it resulted in a minor reduction in the fold change in Sarcomere Shortening from baseline (Figure 5b). The isoprenaline treatment significantly increased Sarcomere Shortening in PH (about 3-fold) and Sham (approximately 2.5-fold) RV cardiomyocytes compared to baseline. In addition, isoprenaline also reduced TTP90 and TTB90 in PH (about 20%) and Sham (about 30%) RV cardiomyocytes. The combination of treprostinil and isoprenaline was unable to induce any significant change in Sarcomere Shortening compared to the isoprenaline effect in PH RV cardiomyocytes. On the contrary, in Sham cells, the combination of treprostinil and isoprenaline was able to induce an increase in fold change in Sarcomere Shortening (3.313 ± 0.222 vs. 2.381 ± 0.117, *p* < 0.05), Δt90 Contraction (0.507 ± 0.028 vs. 0.715 ± 0.056, *p* < 0.05), and Δt90 Relaxation (0.538 ± 0.034 vs. 0.908 ± 0.028, *p* < 0.05) (Figure 5b).

The next set of analyses assessed the effect of the drugs on Cell Length Deflection in MCT-induced PH and Sham RV cardiomyocytes. A similar effect was observed for the treprostinil treatment when comparing Sarcomere and Cell Length Deflection for both PH and Sham cells (Figure 5b,c). The isoprenaline treatment significantly increased the percentage of Cell Length Deflection in PH (2.5-fold) and Sham (2-fold) and reduced TTP90 by 10% in PH and 25% in Sham cells. The TTB90 Cell Length Deflection parameter was not significantly affected by isoprenaline in PH cells, while there was a 25% decrease in Sham cells. The combination of treprostinil and isoprenaline did not significantly affect Cell Length Deflection in PH cells compared to the isoprenaline-induced effect. In Sham cells, the cell response pattern reflected the Sarcomere Shortening response. This was characterised by a significant increase in fold change Cell Length Deflection (3.067 ± 0.125 vs. 2.322 ± 0.172, *p* < 0.01), and a significant reduction in Δt90 Contraction (0.638 ± 0.034 vs. 0.759 ± 0.034, *p* < 0.05) and Δt90 Relaxation (0.602 ± 0.063 vs. 0.750 ± 0.033, *p* < 0.01) when compared to the isoprenaline alone response (Figure 5c). 

**Figure 6:**
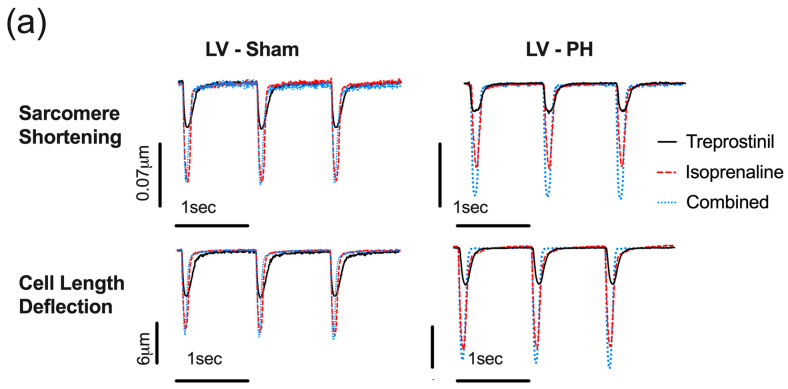

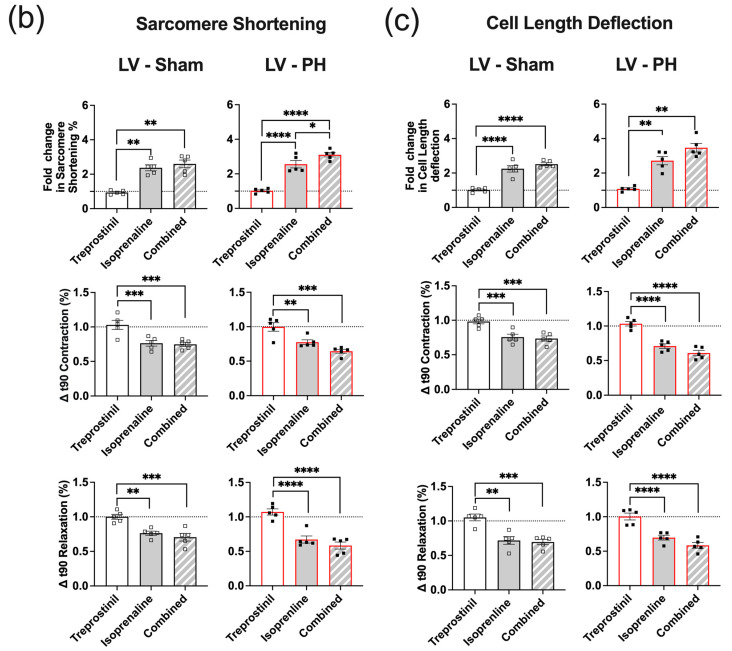
Characterisation of effects of treprostinil, isoprenaline and the combination of treprostinil and isoprenaline on PH (red) and Sham (black) LV cardiomyocytes. Representative traces (**a**) and normalised mean percentage Sarcomere Shortening (**b**) and Cell Length Deflection (**c**). PH animals (black square); Sham animals (white square). Data expressed as mean ± SEM, *n* = 5 rats; * *p* < 0.05, ** *p* < 0.01, *** *p* < 0.001, **** *p* < 0.0001 by nested one-way ANOVA with Tukey’s multiple comparisons.

Next, we assessed the effect of treprostinil, isoprenaline, and the combination of treprostinil and isoprenaline on Sarcomere Shortening and Cell Length Deflection (Figure 6) in PH LV cardiomyocytes compared to Sham. Based on the representative traces, PH LV cardiomyocytes displayed variable responses to treprostinil, isoprenaline, and the combination of both drugs compared to Sham cardiomyocytes (Figure 6a). Based on the normality test, both PH and Sham LV cardiomyocyte populations were normally distributed, so a parametric nested one-way ANOVA was used to compare the response of the cells to the three treatments.

In the first set of analyses, the effect of the agents was assessed on the Sarcomere Shortening of PH LV cardiomyocytes compared to Sham cells (Figure 6b). Based on the results, the treprostinil treatment had no observable effect on PH and Sham LV cardiomyocyte Sarcomere Shortening. The isoprenaline treatment significantly increased Sarcomere Shortening in both PH (about 2.6-fold) and Sham (approximately 2.4-fold) cells compared to baseline. In addition, isoprenaline also reduced TTP90 and TTB90 in PH (about 25% and 35%, respectively) and Sham (about 25%) cells. The combination of treprostinil and isoprenaline was able to induce a significant increase in Sarcomere Shortening (3.105 ± 0.130 vs. 2.558 ± 0.213, *p* < 0.05) and a trend for a reduction in Δt90 Contraction and Δt90 Relaxation (but not significant) when compared to the effect of isoprenaline (Figure 6b). In Sham cells, the combination of treprostinil and isoprenaline was unable to induce any significant change in Sarcomere Shortening compared to the isoprenaline effect. In the next set of analyses, the effect of the drugs was assessed on the Cell Length Deflection in LV cardiomyocytes from PH and Sham animals.

Similar, to the Sarcomere Shortening results, the treprostinil treatment did not affect the Cell Length Deflection for PH and Sham cardiomyocytes (Figure 6a,c). The isoprenaline treatment significantly increased the Cell Length Deflection in PH (2.5-fold) and Sham (2-fold) and reduced TTP90 by 10% in PH and 25% in Sham cells. The TTB90 Cell Length Deflection parameter was not significantly affected by isoprenaline in PH cells, while there was a 25% decrease in Sham cells. The combination of treprostinil and isoprenaline was able to further increase the normalised Cell Length Deflection by 3.5-fold and reduce TTP90 and TTB90 by 40% in PH LV cardiomyocytes. However, the changes were not statistically significant. In Sham LV cardiomyocytes, there was no difference in the Cell Length Deflection parameter between isoprenaline and the combination of isoprenaline and treprostinil (Figure 6c). 

### 3.6. In Established PH RV Cardiomyocytes, Treprostinil-Induced PKA Activation Is Reduced

**Figure 7:**
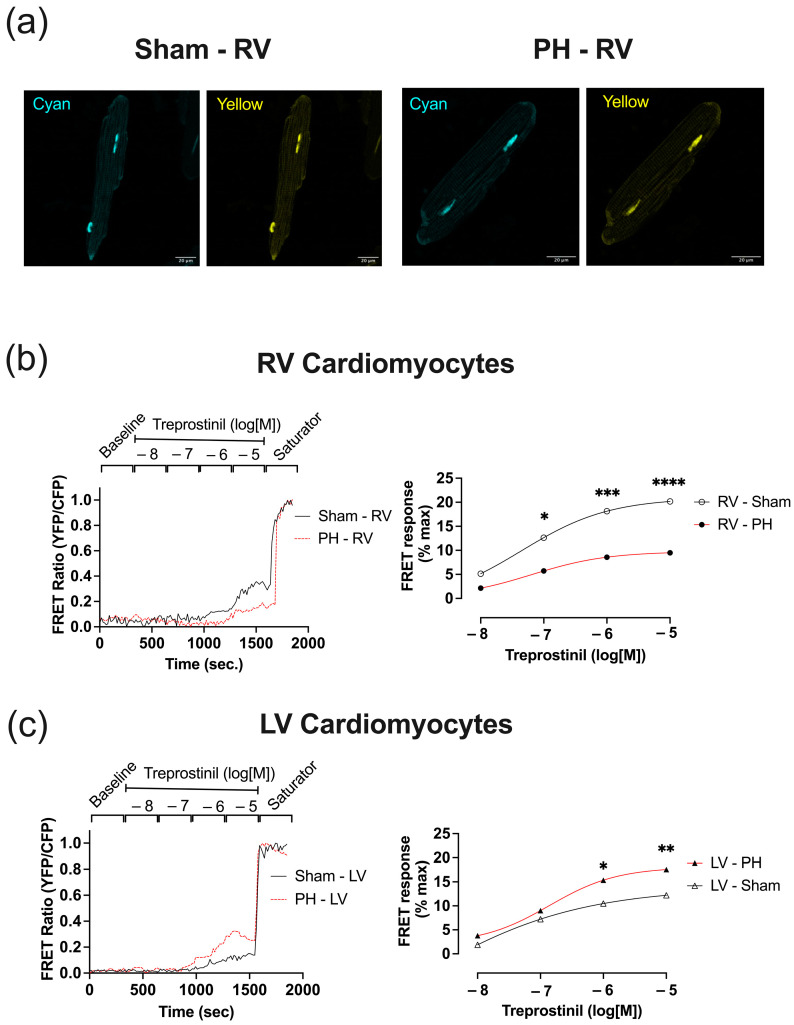
Nuclear FRET responses in PH and Sham ventricular cardiomyocytes expressing AKAR3-NES FRET sensor. (**a**) Pseudo-colour images of RV cardiomyocytes with nuclear localisation of AKAR3-NES FRET-based sensor. Representative normalised FRET curves (Sham—solid black line; PH—red dashed line) and Log(agonist) vs. response Line chart (Sham white symbol/black line; PH—black symbol/red line) to treprostinil (0.01, 0.1, 1 and 10 μM) stimulation followed by Saturator (10 μM Forskolin, 100 μM IBMX) of RV (circle symbol) (**b**) and LV (triangle symbol) (**c**). Data expressed as mean ± SEM; *n* = 30 from 5 animals; * *p* < 0.05, ** *p* < 0.01, *** *p* < 0.001, **** *p* < 0.0001 based on two-way ANOVA with Šídák’s multiple comparisons.

We used a set of FRET-based reporters to measure treprostinil-induced changes in cAMP levels and PKA activity. First, we tested two cAMP sensors: the cytosolic CUTie [34] (Figure S2a) and Epac2-camps [35] (Figure S2b). Both sensors were unable to detect treprostinil-induced changes in cytosolic cAMP activity (Figure S2d). Next, we tested the PKA activity reporters AKAR3-NES (Figure S2c,d) and AKAR3-NLS (Figure 7) [36].

The cytosolic AKAR3-NES reporter was unable to detect treprostinil-induced changes in cytosolic PKA activity (Figure S2d). The AKAR3-NLS FRET-based sensor with a nuclear localised signal (Figure 7) was able to report a change in cAMP/PKA activity in both PH and Sham RV (Figure 7b) and LV cardiomyocytes (Figure 7c). To test if treprostinil-induced PKA activity was affected by the PH pathology, we treated RV and LV cardiomyocytes with incrementally increasing concentrations of treprostinil (0.01 μM, 0.1 μM, 1 μM, and 10 μM) followed by a saturator (100 μM IBMX and 10 μM Forskolin). We showed that at concentrations ranging from 0.1 μM to 10 μM, treprostinil induced significantly lower PKA activity in the nuclear compartment of PH RV cardiomyocytes compared to Sham (Figure 7b). In LV cardiomyocytes, the opposite effect was observed, and based on the representative traces, PH pathology was associated with an increased FRET ratio response (Figure 7c). Based on the normalised FRET ratio response, LV cardiomyocytes from PH animals exhibited a significantly greater nuclear response at doses of 1 μM (15.350 ± 1.833 vs. 10.480 ± 0.914, *p* < 0.05) and 10 μM (17.560 ± 2.004 vs. 12.200 ± 0.913, *p* < 0.01) compared to Sham LV cardiomyocytes (Figure 7c).

## 4. Discussion

In this study, we observed that the induction of PH with MCT impaired cardiomyocyte contraction and disrupted the synergistic effect of treprostinil and isoprenaline. We successfully demonstrated that 3 weeks post-MCT, the RV undergoes decompensation. We confirmed that MCT-treated PH animals had decreased BW and exhibited both lung and heart remodelling, consistent with the prior research [20,21]. PH RV cardiomyocytes also exhibited structural changes, including sarcomere irregularity and hypertrophy, indicative of maladaptive hypertrophic remodelling [13,22,27]. The comparison of the Sarcomere Shortening and Cell Length Deflection indicated a loss of the Cell Length Deflection lusitropic effect in PH, indicating impaired RV cardiomyocyte relaxation and suggesting pathology-associated impairment in the contractile machinery or an alteration in the basal Ca^2+^ load [37,38]. 

Consistent with previous studies, our observations revealed that PGI_2_ analogue treprostinil and catecholamine isoprenaline together produce a synergistic effect [28,39] characterised by enhanced contractility. However, this effect was not observed in the RV cardiomyocytes from PH rat. First, we performed contraction experiments with isolated RV and LV cardiomyocytes from PH and Sham rats stimulated with treprostinil, isoprenaline, or the combination of the two drugs. Treprostinil on its own had a negligible effect on cardiomyocyte contraction. The status of PGI_2_ and its mimetics as modulators of cardiac physiology and cardiomyocyte contraction remains controversial in the scientific community, with limited evidence of direct inotropic effects in the whole heart, cardiac tissues, and isolated cardiomyocytes [28,40,41,42,43]. One study using isolated hearts attributed an inotropic effect of treprostinil and MRE-269 (a selective agonist of the IP receptor) to vasodilation of the coronary arteries in the heart. The same study also reported a compensatory mechanism associated with IP receptor expression and its loss in failing hearts [44].

A standard dose of isoprenaline [26] induced classical inotropic and lusitropic responses in RV and LV cardiomyocytes from both PH and Sham animals and a minor reduction in the response of PH cells, which is consistent with catecholamine desensitisation in heart failure [45]. Isoprenaline activates both β_1_-AR and β_2_-AR receptors, which are associated with distinct nanodomain cAMP pools and contribute to positive inotropic and lusitropic effects [46,47]. In healthy cardiomyocytes, cAMP from β_2_-AR is known to be localised to t-tubules, while the progressive disorganisation of cellular structures in pathologies shifts receptor expression to different cell surface domains [35]. In the setting of LV myocardial infarction, β_1_-AR, but not β_2_-AR, was shown to contribute to cardiac dysfunction; therefore, in the future, it would be interesting to investigate the effect of established PH pathology on the localisation and function of selective β-AR subtypes [46,47,48]. 

The synergistic effect of a combination of treprostinil and isoprenaline was previously reported by many studies in cardiac tissues and cells [28,49,50,51,52,53]. We wanted to investigate the effect in the setting of established PH-associated RV maladaptive remodelling. PH RV cardiomyocytes failed to show enhanced contractility when subjected to the combination of the two drugs. The functional association of the recently described β-AR subtype-specific nanodomain-associated intracellular cAMP pools could be responsible for the PGI_2_–catecholamine crosstalk. The disorganisation of cardiomyocyte structure might result in receptor re-localisation and cause signal disruption [35,46,47].

Previously, some studies attributed the positive inotropic effect of PGI_2_ to the release of tissue catecholamines, which is blocked by propranolol, a conventional β-AR blocker [51,52] and the synergistic effect of treprostinil and isoprenaline was reported on cell length shortening and to a lesser magnitude on isolated Langendorff hearts [28]. These studies did not report any changes in the speed of contraction or relaxation due to treprostinil on isolated cardiomyocytes, an effect that we were able to demonstrate. 

We identified an uncoupling of cellular relaxation in RV cardiomyocytes. This pathology is specific to this cell type in PH. Our group previously reported that remodelling of the cardiomyocyte t-tubule system begins at an early PH stage [22]. In this study, we did not look at the t-tubule structure, only at the sarcomere organisation [25,27]; still, the two structures are linked, and a reduction in sarcomere regularity in the PH RV cardiomyocytes was associated with a maladaptive remodelling phenotype. This may contribute to a decreased efficacy of the transduction of Sarcomere Shortening to Cell Length Deflection and Sarcomere Relaxation to Cell Length Relaxation through the contractile machinery.

We also identified a PH-associated reduction in Ca^2+^ transients. The detrimental effect of PH on RV cardiomyocyte sarcomere organisation and contraction at baseline and following isoprenaline stimulation were also reflected in reduced CaT amplitudes. Reduced CaT amplitudes were previously reported in isolated failing porcine and human LV myocytes and RV myocytes from early-stage MCT-induced PH [54,55]. The loss of t-tubule density in adult ventricular cardiomyocytes, and the dysregulation of calcium transients and cardiomyocyte contraction occurred concomitantly [13].

Finally, we observed a PH pathology associated with RV cardiomyocytes, which reduced treprostinil-mediated PKA activity. This study used a set of FRET-based cAMP and PKA sensors to measure treprostinil-induced responses. We have used Epac2-camps [35] and cytosolic CUTie [34] as cAMP sensors and AKAR3-NES and AKAR3-NLS and PKA sensors [36] previously and extensively for cardiomyocyte physiology experiments. Interestingly, we were unable to detect treprostinil-associated cAMP and PKA changes in RV cytosolic compartments. However, we were able to detect treprostinil-induced changes in Sham RV cardiomyocytes’ nuclear PKA activity.

Furthermore, we observed a reduction in treprostinil activity in failing RV cardiomyocytes from PH animals, consistent with the contraction data. There was also an elevation of the treprostinil-associated response in PH LV cardiomyocytes, consistent with the contraction results and suggesting a potential compensatory mechanism that needs further study. The PH-associated reduction in the treprostinil-induced response in RV cardiomyocytes could be attributed to the loss of t-tubules and impaired signal transduction to the nuclear compartment. It could also be associated with alterations in AKAPs and PDE activity. 

Previously, a study using AKAR3-NES and AKAR3-NLS in rat ventricular cardiomyocytes reported a β-AR subtype selectivity in nuclear PKA activation [56]. Previous studies have reported that the augmentation of excitation–contraction coupling in stimulated cardiomyocytes is attributed to PDE activity modulation. Selective PDE inactivation prevents the hydrolysis of specific cAMP pools. β-AR stimulation-induced local cAMP elevation activates PKAs and modulates the contractile machinery via protein phosphorylation [57]. The activity of treprostinil may thus be attributed to PDE modulation and in combination with β-AR receptor stimulation enable to surpasses a cAMP threshold and alter the contractile properties of the cells. 

The proposed mechanism of action of maladaptive remodelling in the MCT model of PAH is that the increased afterload experienced by a heart will result in compensatory hypertrophy. This immediate adaptation is beneficial and serves the purpose of preserving the contractile function of the heart. Due to the compliant nature and poor adaptive potential of the RV, a chronic increase in afterload can result in quick progression to cardiac decompensation. In addition, the sympathetic hyperactivity essential to maintaining cardiac output in this pathological state will cause cardiomyocyte apoptosis and maladaptive tissue remodelling, further exacerbating the pathology.

## 5. Conclusions

This study has provided further evidence of the potential synergistic interplay between PGI_2_ and β-AR pathways in cardiomyocytes and indicated a possible contribution of heart remodelling in the PGI_2_ therapy desensitisation observed in patients. The current guidelines advise against using β-blockers in PAH patients [58]; however, a synergistic interaction between PGI_2_ and β-AR pathways suggests further research is required to understand if any PAH patient subgroup might benefit from β-blockers. 

We have yet to investigate the effect of the drug combination on early-stage PAH with cardiac compensation. However, our results indicate that treprostinil can act as a potent amplifier of catecholamine effects, so further research is necessary to understand its potential contribution to hyperactivated sympathetic system activity in the transition from compensatory to maladaptive remodelling. Further studies with compensating cells, β-AR blockers, and PDE inhibitors are required to fully comprehend the mechanism of β-AR and PGI_2_ pathway interactions in adult healthy and PH RV cardiomyocytes.

This study has several limitations associated with the research. In the current study, male rats were utilised to understand the basic cellular mechanism of ventricular cardiomyocyte maladaptive remodelling and the associated changes in treprostinil pharmacology. In the future, it will be essential to investigate PH-associated ventricular cardiomyocyte remodelling in large animal models and a female population. Furthermore, this study utilised an established PH model, and to fully appreciate the temporal change in treprostinil pharmacology, early- and late-stage disease should be assessed, which would allow us to understand if RV desensitisation is directly associated with pressure overload. No assessment of the possible change in high-affinity treprostinil receptor expression was conducted.

## Figures and Tables

**Table 1:** Quantification of RV and LV cardiomyocyte calcium transients and contraction from pulmonary hypertension (PH) and Sham right (RV) and left ventricular (LV) cardiomyocytes. Data presented as mean ± SEM, *n* = 5; * *p* < 0.05, ** *p* < 0.01, *** *p* < 0.001 by nested *t*-test, n.s.: no significance.

Chamber	Sham (Mean ± SEM)	PH (Mean ± SEM)	*p*-Value Hierarchical
Calcium Transients Amplitude (Fura-2AM ratio)			
RV	0.031 ± 0.005	0.020 ± 0.003	0.0108 (*)
LV	0.037 ± 0.003	0.030 ± 0.003	0.2046 (n.s.)
Calcium TTP90 (sec.)			
RV	0.028 ± 0.002	0.034 ± 0.003	0.0231 (*)
LV	0.027 ± 0.002	0.032 ± 0.003	0.1853 (n.s.)
Calcium TTB90 (sec.)			
RV	0.300 ± 0.010	0.409 ± 0.040	0.0063 (*)
LV	0.301 ± 0.025	0.359 ± 0.014	0.1416 (n.s.)
Sarcomere Shortening (%)			
RV	3.899 ± 0.285	2.82 ± 0.2048	0.0279 (*)
LV	5.259 ± 0.562	4.057 ± 0.5356	0.1311 (n.s.)
Sarcomere Shortening TTP90 (sec.)			
RV	0.049 ± 0.003	0.060 ± 0.006	0.0145 (*)
LV	0.050 ± 0.004	0.057 ± 0.003	0.0238 (*)
Sarcomere Shortening TTB90 (sec.)			
RV	0.193 ± 0.024	0.272 ± 0.025	0.0096 (**)
LV	0.222 ± 0.019	0.266 ± 0.018	0.0348 (*)
Cell Length Deflection (%)			
RV	3.702 ± 0.250	2.238 ± 0.387	0.0003 (***)
LV	5.565 ± 0.782	4.541 ± 0.461	0.065 (n.s.)
Cell Length Deflection TTP90 (sec.)			
RV	0.050 ± 0.002	0.063 ± 0.004	0.0009 (***)
LV	0.047 ± 0.003	0.055 ± 0.005	0.0084 (**)
Cell Length Deflection TTB90 (sec.)			
RV	0.224 ± 0.017	0.328 ± 0.045	0.0026 (**)
LV	0.250 ± 0.031	0.258 ± 0.012	0.6909 (n.s.)

## Data Availability

All data are available from corresponding author upon reasonable request.

## Supplementary Materials

The following supporting information can be downloaded at: https://www.mdpi.com/article/10.3390/cells12232764/s1, Figure S1: Proportion of arrhythmic cells in pulmonary hypertension (PH) ventricular cardiomyocyte populations; Figure S2: Expression of cytosolic CUTie.
