# Supplementary material for: Pulmonary Hypertension-Associated Right Ventricular Cardiomyocyte Remodelling Reduces Treprostinil Function
Source: Cells. 2023 Dec 4;12(23):2764. doi: 10.3390/cells12232764 (PMC10705885; doi:10.3390/cells12232764)
Supplement: Supplementary file 1 [file cells-12-02764-s001.zip › cells-2713448-supplementaryl.pdf]

Supplementary Figures

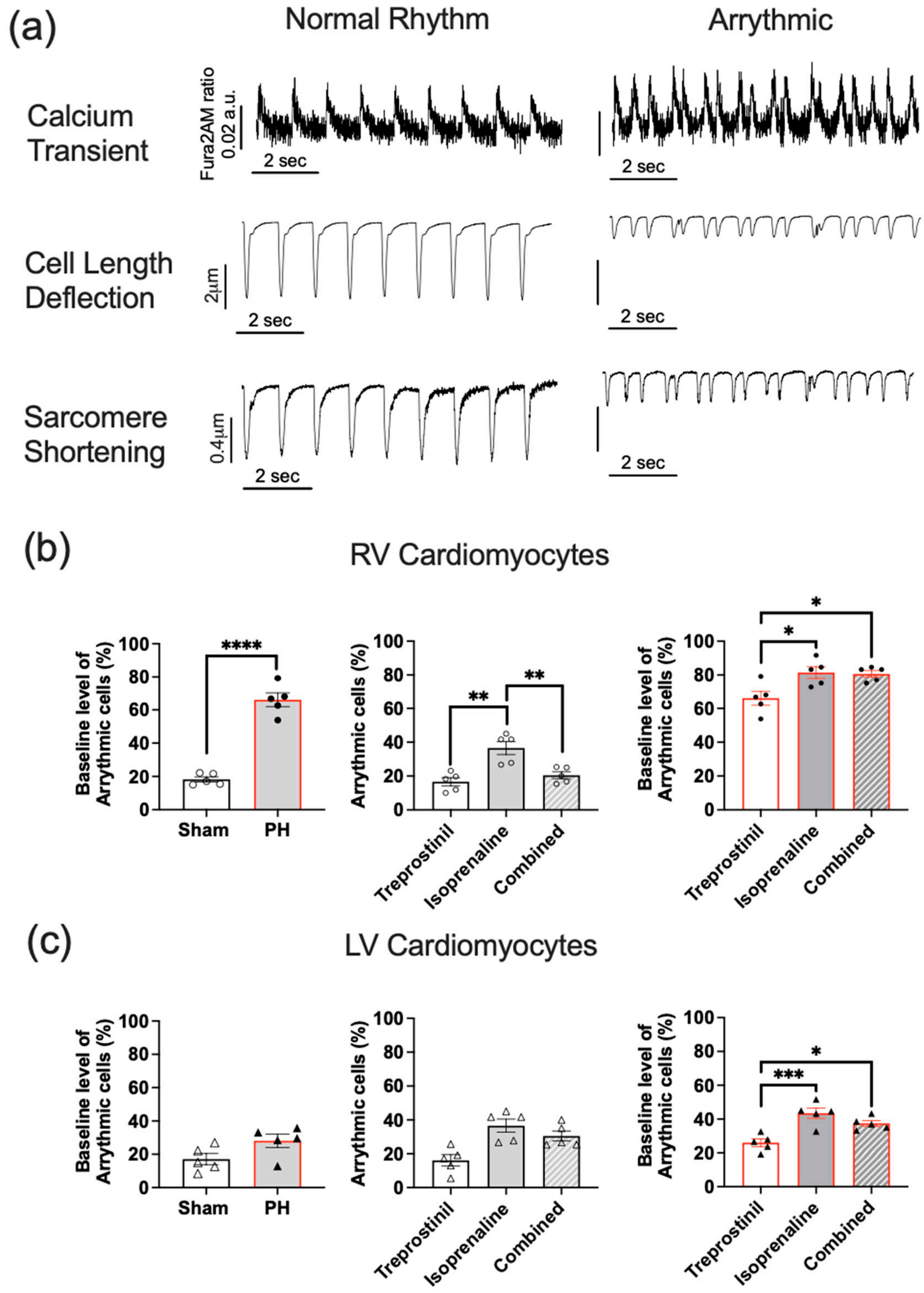

Figure S1. Proportion of arrhythmic cells in pulmonary hypertension (PH) ventricular cardiomyocyte populations. (a) Representative traces of paced ventricular cardiomyocyte Ca^{2+} Transient, Sarcomere Shortening and Cell Length Deflection with Normal Rhythm and arrhythmic phenotype. The proportion of RV (b; circle) and LV (c, triangle) arrhythmic cells at baseline and following drug treatment in PH (red; white figure) and Sham (black; black figure). Data expressed as mean ± SEM; *n* = 5 rats; * $p < 0.05$, ** $p < 0.01$, *** $p < 0.001$, **** $p < 0.0001$) by Nested 1 -way ANOVA with Tukey's Multiple Comparison.

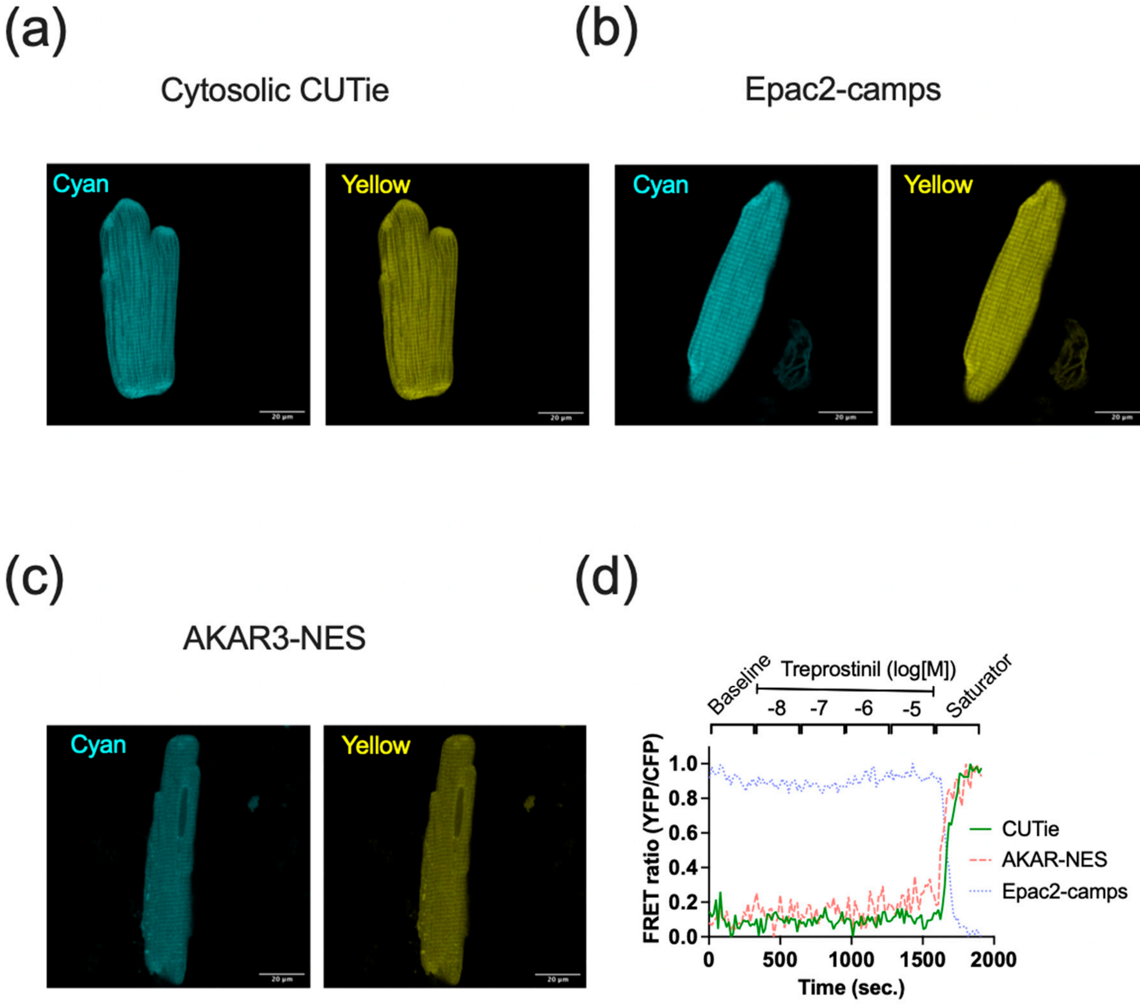

Figure S2. Expression of Cytosolic CUTie (a), Epac2-camps (b) and AKAR3-NES (c) FRET based sensors and Treprostinil induced changes in cAMP levels (d) in rat adult RV cardiomyocytes.
